# Statistical Power Analyses for Quantifying the Similarity of Categories of Surgical Procedures Among Pairs of Hospitals and Ambulatory Facilities

**DOI:** 10.7759/cureus.81761

**Published:** 2025-04-05

**Authors:** Franklin Dexter, Richard H Epstein, Richard P Dutton, Rachel A Hadler

**Affiliations:** 1 Anesthesia, University of Iowa, Iowa City, USA; 2 Anesthesiology, University of Miami Miller School of Medicine, Miami, USA; 3 Anesthesia, United States Anesthesia Partners, Dallas, USA; 4 Anesthesiology, Emory University, Atlanta, USA

**Keywords:** hospital management system, industrial engineering, management science, managerial epidemiology, mixed methods, organizational behavior

## Abstract

Introduction: Mixed methods are often used to understand organizational associations and differences. For example, one might compare hospitals and ambulatory surgery centers, each described by its relative distribution of cases’ categories of surgical procedures, quantified using anesthesia Current Procedural Terminology (CPT) codes. The similarity of these distributions between facilities can be assessed using a metric akin to a correlation coefficient. Conceptually, identifying similar organizational pairs is feasible, as most U.S. states and Canadian provinces maintain databases containing such administrative data. However, research proposals based on mixed methods may be hindered by the lack of statistical power analysis to determine whether the quantitative phase will yield a sufficient number of similar facilities to support the qualitative phase (i.e., interviews).

Materials and methods: Data were obtained from the American Society of Anesthesiologists' National Anesthesia Clinical Outcomes Registry. The dataset included 12,902,159 cases across 272 procedure categories, performed at 2442 facilities in the United States. The similarity index between facilities ranged from 0 (no overlap in surgical procedures) to 1 (identical distribution of procedures). Values ≥0.80 were considered indicative of high similarity. We estimated the proportion of highly similar facility pairs (similarity index ≥0.80) with low standard errors (<2.0). For each pair, we computed the inverse of the standard normal distribution based on the ratio of the difference from 0.80 to the standard error. The average of these values yielded the mean prevalence of high similarity. This estimated prevalence was then used in power analyses based on the binomial distribution.

Results: Only 1.00% (standard error: 0.01%) of facility pairs had a similarity index ≥0.80. Based on this prevalence, a database would need to include just 38 organizations to have an ≥80% probability of identifying at least five highly similar pairs for interviews. With data from 67 organizations, there would be a ≥95% probability of identifying at least 15 pairs. In contrast, consider an individual organization deciding whether to (a) join a consortium to identify similar organizations for shared strategies, or (b) invest in analysts to explore mandatory state or provincial databases for such purposes. Unless more than 1,000, and ideally more than 2,100, organizations contribute data, the probability of finding multiple highly similar peers may be low.

Conclusions: Investigators can expect a high probability of obtaining sufficient organizational sample sizes for qualitative interviews when using large-scale databases. Although only a small fraction (approximately 1%) of organization pairs exhibit high similarity, the sheer number of potential pairs in state, provincial, and national databases compensates for this. However, for an individual organization seeking to identify peers for qualitative comparison, the chance of finding highly similar matches based on similar surgical procedures is extremely low, unless joining a very large data collective.

## Introduction

Mixed methods synthesize quantitative data analysis and qualitative information (e.g., interviews). Mixed methods are often used to understand associations and differences among organizations. For example, consider comparing hospitals and ambulatory surgery centers, each described by its relative distribution of cases’ categories of surgical procedures, quantified using the anesthesia Current Procedural Terminology (CPT) codes. The similarity of the relative distribution of procedures can be compared between facilities, like a correlation coefficient [1,2]. Applications include comparing staff scheduling approaches for nights and weekends [3], clinicians’ training and specialization [4], hospitals’ care of specialty patients [5,6], clinical workflows [7], and strategic positioning of the organization relative to competitors [8,9]. Leaders and managers can then be interviewed to compare paired organizations.

Conceptually, finding pairs of organizations is not a limitation, as most states in the United States and provinces in Canada have databases with these administrative data [2,4-9]. Research and quality databases such as the American Society of Anesthesiologists (ASA) National Anesthesia Clinical Outcomes Registry (NACOR) can also be used [3]. However, research proposals centered upon mixed methods can be stymied by the absence of statistical power analysis to assess whether the quantitative analysis will yield enough similar facilities to complete the qualitative portion of the study (i.e., the interviews). The singular aim of our study was to develop such a methodology; there were no secondary objectives. An earlier anesthesia review on similarity methods for comparing procedures did not include sample size selection [2] because there were no relevant papers, and no such study has been conducted. (Stopping rules are available for prospective observational studies with progressive assessments of similarity until known with sufficient precision [10], not applicable to our consideration of large retrospective cohorts for selecting organizations for qualitative study.)

## Materials and methods

The University of Miami Human Subject Research Office determined that this project is not human subject research requiring institutional review board review, approval, or oversight. The study does not involve interactions with living human subjects or accessing identifiable information or identifiable biospecimens. The study does not meet the definition of a human subject found at 45 CFR 46.102(e). This study was performed following a data use agreement with the ASA to use their deidentified NACOR data [11]. Anesthesia CPT codes are "00100" through "01999". For example, "01961" is "anesthesia for cesarean delivery only," and "00580" is "anesthesia for heart transplant or heart/lung transplant."

Our retrospective cohort study used the 16,328,705 records from two studied years, 2022-2023. Cases removed were the 1,756,862 with missing facility, 1,219,924 with missing start or end time, 447,903 with no anesthesia CPT code, and 1857 from facilities, each with <12 cases total over the two years. This resulted in 12,902,159 cases (79%) from 2442 facilities. With 272 categories of procedures, there were 664,224 combinations of facility and procedure, mostly zeros.

The blinded facility identifiers were renumbered 1, 2, …, 2442. All possible distinct pairwise combinations of the numbers 1, 2, …, 2442 were listed in one column’s successive rows (e.g., 1, 2, 1, 3, 1, 4, …, 2441, 2442). Then, the original file with the counts of cases for each of the 272 anesthesia CPTs was merged. Similarity indices were calculated between successive rows (e.g., first and second rows for the first and second facilities and third and fourth rows for the first and third facilities). There were 2,980,461 estimates of similarity, where 2,980,461 = 2442 × (2442 - 1) / 2.

The similarity index (θ) was calculated using the formula described in Yue and Clayton [1] and Dexter et al. [2]. Specifically, envision selecting a case randomly from all the cases performed at a facility [1,2]. Then, select a case from the other facility [1,2]. Repeating that process many times, the probability that selected cases’ anesthesia CPTs were the same is the numerator of the similarity index [1,2]. The denominator normalizes the range from zero (when there is no overlap of surgical procedures ) to one (when the distribution of procedures among the cases is the same for the facilities) [1,2]. The full formula is Dexter et al.'s [2] equation (8). Relatively large similarity index values are θ≥0.80 [2,3,7]. Similarity index values 0.30≤θ≤0.80 are considered “moderate” [2,3,7]. The standard error of the similarity index was calculated using the equations listed as equation (20) in the earlier statistical grand rounds on the topic [2].

To estimate the mean proportion of the similarity indices that were large (θ≥0.80), we calculated the inverse of the standard normal distribution for each pair’s ratio of the difference from 0.80 and the standard error of the estimate. The average of the inverses gave the mean prevalence of θ≥0.80. The standard error of the mean was calculated analytically using the standard deviation and counts of the pairs' ratios.

## Results

**Figure 1:**
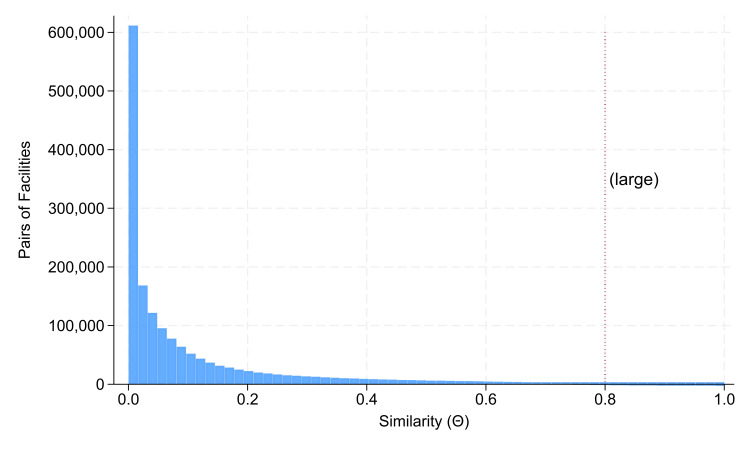
Histogram of the similarity index (θ) between the categories of surgical procedures at pairs of facilities From the N=2442 facilities, there were 2,980,461 pairs of facilities, where 2,980,461 = 2442 × (2442 - 1) / 2. Among those pairs, there were 1,673,534 pairs with a small standard error of the similarity index (<0.02), and both facilities had two or more categories of procedures performed commonly, based on the inverse of each facility’s internal Herfindahl. The figure shows a histogram of the 1,673,534 estimates of similarity between the facilities’ procedures. Over the two years, 2022-2023, there were 272 such anesthesia Current Procedural Terminology categories of codes with at least one case. Several features of the monotonic decrease in the frequency of similarity indices (θ) among pairs of facilities are important mathematically. First, the most commonly observed frequency was θ=0.00. In other words, it was not 0.00<θ<1.00, but 0.00≤θ≤1.00. That relationship rules out multiple probability distributions. Second, the standard deviation of 0.172 is considerably larger than the mean of 0.113, unlike for the exponential distribution. Third, there were 1.00% (standard error 0.01%) of the similarity index estimates θ≥0.80 (i.e., large).

Among the 2,980,461 pairs of facilities’ procedures, there were 1,673,534 with a small (<2.0) standard error of the similarity indices, and both facilities had at least two categories of procedures performed commonly, based on the inverse of the Herfindahl (i.e., the sum of the squared proportions attributable to each procedure) [2,3,5-7,9]. Figure 1 shows a histogram of the 1,673,534 estimates of similarity. The mean prevalence of θ≥0.80 equaled 1.00% (standard error 0.01%). When repeated with no restrictions on the standard error or the internal Herfindahl (i.e., diversity) of procedures at the two facilities (i.e., with all 2,980,461 pairs), the mean prevalence of θ≥0.80 equaled 1.50% (standard error 0.01%). These prevalences are our report’s novel results.

Statistical power analyses were performed using the observed 1.00% prevalence. Table 1 shows that there would need to be only 38 organizations in a database to have ≥80% probability of obtaining at least five pairs with large similarity (θ≥0.80) for interviews. With only 67 organizations’ data, there would be ≥95% probability to obtain ≥15 pairs. This was because N=67 yields 2211 potential pairs, where 2211 = 67 × (67 - 1) / 2. The 15/2211 is less than 0.01. In contrast, consider an individual organization contemplating either (a) joining a consortium in part to know of similar organizations to share strategies or (b) investing in analysts to use mandatory databases (e.g., state or province) for such purposes. Table 1 shows that unless there are >1000 organizations contributing data, and ideally >2100 organizations, the probability of finding several organizations with large similarities may be low.

**Table 1:** Smallest database size (N) of organizations to achieve desired statistical power (β) based on 1.00% prevalence of pairwise similarities θ≥0.80 ^a^ Calculations can be repeated easily with any other prevalence. For example, to do so in Stata using the largest expected prevalence of 1.5% (see results) and for the first cell of this table, find the smallest N such that binomialtial (N, 5, 0.015) ≥ 0.80. The "binomialtial" is the Stata v18.5 command (StataCorp, College Station, Texas). ^b^ As context, 38 organizations have 703 case pairs, where 703 = 38 × (38-1) / 2. ^c^ As context, 67 organizations have 2211 case pairs, where 2211 = 67 × (67-1) / 2.

Database characteristics for mixed methods based on 1.00%^a^ prevalence	≥80% probability (β≤0.20)	≥90% probability (β≤0.10)	≥95% probability (β≤0.05)
≥5 pairs with similarity index θ≥0.80, for 10 assessments of practices	38^b^	41	44
≥10 pairs with similarity index θ≥0.80, for 20 assessments of practices	51	54	57
≥15 pairs with similarity index θ≥0.80, for 30 assessments of practices	61	64	67^c^
≥5 organizations to be paired with one target organization based on similarity index θ≥0.80, for six assessments of practices	672^b^	799	914
≥10 organizations to be paired with one target organization based on similarity index θ≥0.80, for 11 assessments of practices	1252	1419	1569
≥15 organizations to be paired with one target organization based on similarity index θ≥0.80, for 16 assessments of practices	1812	2011	2186^c^

## Discussion

Our power analysis study shows that mixed methods can be highly effective for comparing similar organizations based on categories of surgical procedures [7]. Table 1 shows that investigators should expect a high probability (e.g., ≥95%) of having sufficient sample sizes of organizations for interviews. Few (≈1.00%) of the pairs of organizations have large similarities (θ≥0.80). By our having estimated that "few" means ≈1.00%, because many thousands of pairs are examined, the probability of having enough pairs of organizations for interviews is high. Therefore, state, provincial, and national databases can be used to choose pairs for qualitative analyses.

Our results also show a negligible value for all but the largest collectives to provide meaningful pairings for individual prespecified organizations. Most importantly, Figure 1 shows that the premise of envisioning that participating in some group to have some prespecified qualitative “peers,” sufficiently similar in procedure distributions to justify qualitative comparison, is in reality extremely low, regardless of whether to compare staff scheduling at nights and weekends [3], clinicians’ training and specialization [4], care of pediatric patients [5,6], operational workflows [7], or strategic planning [8,9].

We explained in the Introduction that an earlier anesthesia review on similarity methods for comparing procedures did not include sample size selection [2] because there were no relevant papers, and no such study has been conducted. Again, as explained there, stopping rules are available for prospective observational studies with progressive assessments of similarity until known with sufficient precision [10]. However, those do not apply to our consideration of large retrospective cohorts for selecting organizations for qualitative study. Having created Table 1, we repeated searches using Scopus on March 13, 2025. There were no matching documents for TITLE ("statistical power" OR "power analys*") AND TITLE (similarit*) AND TITLE (prevalence). Excluding the last term, two documents were returned, neither relevant. Repeating instead using TITLE-ABS, three documents were returned, but again, none were relevant. We performed these searches in Scopus because many journals publishing operating room management science are not in PubMed [12]. Since 2020, fewer than half of the new operating room management articles have been published in PubMed because most are in engineering journals [12]. We repeated our search using Web of Science, the source of the other recent bibliometric study of operating room management [13]. There were seven documents returned using TS=("statistical power" OR "power analys*") AND TS=(similarit*) AND TS=(prevalence), none relevant. These Scopus and Web of Science search findings highlight the originality of our research.

The principal limitation of our results is that they are based on pairwise comparisons of organizations. Specifically, the similarity index compares pairs (Figure 1) [1-8]. The statistical power analysis applying the 1.00% prevalence (Table 1) is based on pairs. Considerations of studies involving multiple similar hospitals (e.g., three control organizations for each one study organization, all highly similar to one another) would be less practical.

## Conclusions

Research proposals centered upon mixed methods to compare organizations were limited by the absence of a statistical power analysis method to assess whether there would be enough similar facilities to complete the qualitative interview part of the study. Using a database with millions of pairs of facilities with anesthetics, we showed that the prevalence of large (θ≥0.80) pairwise similarity was ≈1.00%. Using that result in power analysis calculations shows that even as few as 67 organizations’ data would be expected to assure ≥95% probability of obtaining ≥15 pairs for a qualitative study. We look forward to this result being used for the design of future database studies.
